# Notes on Preparations for the Sick

**Published:** 1904-03

**Authors:** 


### Notes on Preparations for the Sick.

Messrs. Burroughs, Wellcome & Co. announce that a **Historical Medical Exhibition** of rare and curious objects relating to Medicine, Chemistry, Pharmacy, and the allied sciences is to be held in London in 1904, in commemoration of the foundation of their firm. Mr. Henry S. Wellcome states that the exhibition will be strictly professional and scientific in character; the intention is to bring together a collection of historical objects illustrating the development of the art and science of healing throughout the ages. He asks for the assistance of all who possess objects within the range proposed, which is a very wide one, embracing paintings, drawings, engravings, photographs, medallions, sculptures, manuscripts, books, letters, diplomas, instruments, apparatus, and curiosities of medicine, surgery, pharmacy, and allied sciences.

The historical souvenir of the British Medical Association at Swansea last year, entitled *Antient Cymric Medicine*, is an indication of Mr. Wellcome's enthusiasm and industry in the collection of archæological records of medical and surgical interest. We have no doubt that the proposed exhibition will be worthy of and will do credit to its promoters.

---

**Tabloids.**—BURROUGHS, WELLCOME & CO., London.—*The Tabloid Case.* The proprietary rights of this firm to the name Tabloid have been amply established by the action brought by this firm against Messrs. Thompson and Capper, of Manchester. After a seven days' trial judgment was given in favour of Burroughs, Wellcome & Co. on all counts. The sin of substitution has been fully exposed, and the medical profession may have increased confidence that they may depend upon the faithful dispensing of prescriptions. It is a little unfortunate, perhaps, that the form of the drug becomes as important, or more so, than the drug itself, and that proprietary rights should enter into the prescription at all; but we have to accept the fact that these rights must be respected if we depart from the limited range of the Pharmacopœia. Of one thing we ought to be sure, that if a proprietary article is prescribed that that alone, and not something like it, is to be dispensed.

The following tabloids have recently been forwarded to us as new preparations:—

Tri-bromides Effervescent.—Each tabloid contains approximately 15 grains of the mixed bromides of potassium, sodium, and ammonium in a soluble effervescing form. Such a combination is convenient, and may sometimes be more useful than the corresponding dose of any one of these bromides.

Hydrarg.-Perchlor., gr. $\frac{1}{32}$, et Potassii Iodidi, gr. ijss.—This tabloid is half the strength of one which was issued some time since. It may be useful when it is needful to continue the administration of these drugs in small doses for a long period.

Antifebrin Compound—Antifebrin, gr. ij.; caffeine citrate, gr. j.; camphor monobromide, gr. j.—This combination is useful in various forms of headache and neuralgic affections. It may be prescribed in all cases where its principal ingredient, acetanilide, is indicated.

Sparteine Sulphate, gr. j.—This drug is often useful as a cardiac tonic and diuretic.

Zinc Valerianate, gr. ij.—These sugar-coated tabloids are readily soluble, and have none of the unpleasant features of the crude drug.

---

**Cyllin (Disinfectant), Cyllin (Medical) Fluid.**—JEYES' SANITARY COMPOUNDS COMPANY, 64 Cannon Street, London.—In our last number (Vol. xxi., p. 375) a notice of Creolin and Creolin (Medical), we are now informed that having regard to the confusion referred to by Dr. Rideal in his essay appearing in *Public Health* for December, 1903, entitled "Creolin: A Note of Warning," in reference to various inferior products of different compositions sold under the name of "Creolin," the Jeyes' Sanitary Compounds Company, Limited, who are the sole owners of that mark in Great Britain and British Colonies, and have the exclusive right of sale of "Creolin" therein, beg to announce that from and after January 1, 1904, the liquid products now sold by them as "Creolin" (Disinfectant) and "Creolin" (Medical) will be labelled and sold as "Cyllin" and "Cyllin" (Medical). The company have accordingly registered the word "Cyllin" as their trade mark.

---

**Pepule—Pepsin and Zymine.**—FAIRCHILD BROS. AND FOSTER, London.—These sugar-coated pepules contain two grains of pepsin and three of zymine, containing the three pancreatic enzymes. The combination may be useful in many cases of indefinite dyspeptic troubles, when it is difficult to determine which of the two preparations is likely to be the more useful.

---

**Pure Sterilized Cream.**—FUSSELL AND CO., 4 Monument Street, London.—This sample was quite like ordinary fresh cream: there was nothing unpleasant in its flavour, and it appeared to be everything it is represented to be.

---

**Hopogan (Powder or Tablets):** Peroxide of Magnesium, $MgO_2$. **Ektogan:** Peroxide of Zinc, $ZnO_2$.—THE BRITISH DRUGGISTS, LTD., London.—These peroxides are of some chemical interest, and should be of therapeutic value. Their names do not connote anything of their nature or properties, but chemical examination shows that Hopogan contains a large amount of magnesium peroxide and Ektogan a large amount of zinc peroxide. Both substances will give hydrogen peroxide first and then oxygen when brought into contact with organic matter.

If the oxide of magnesium be taken internally, hydrogen peroxide will be at once formed in the stomach and at once decomposed, giving nascent oxygen. It should therefore be of use whenever increased oxidation and antisepsis of the stomach are desirable.

The zinc peroxide should act locally as an antiseptic, owing to the liberation of nascent oxygen from contact with organic matter. The Ektogan gauze and Ektogan salve should be useful for surgical purposes; the powder unmixed may be used for erosions of skin and mucous membranes.

---

**Lithiated Sorghum Compound; Mel-Maroba; Ergotole.**—Sharp and Dohme, Baltimore.—These manufacturing chemists of the United States have forwarded through Messrs. Thomas Christy and Co., of London, the above-named "meritorious specialities."

The Lithiated Sorghum Compound is described as a palatable demulcent diuretic, containing broom-corn seed, corn silk, saw palmetto, hydrangea, and lithium bromo-citrate. The broom-corn seed gives its name (Sorghum saccharatum) to the compound, which, from its elaborate composition, should be most effective as an antiseptic diuretic. It is difficult to understand how such a compound can be all which its authors claim, but "whenever it is desirable to use a tonic, stimulating, sedative-to-the-mucous-membrane diuretic," this is the combination to employ.

The Mel-Maroba is called an ideal tonic-alterative. It contains manaca, caroba, stillingia, with 16 grains of potassium iodide in each fluid ounce. Its utility is obvious.

The Ergotole is an assayed, purified, permanent, liquid preparation, each minim representing 2½ grains of select Spanish ergot. It is the standard ergot of America.

---

We have received samples of some of the **Newer Products** of Messrs. Parke, Davis, & Co., London:—

**A Pocket Atomiser and Nebuliser, No. 38** (Parke Davis) is an exceedingly neat and effective spray for oily solutions, and as it can be carried in the waistcoat pocket it will be found very useful in cases where frequent intra-nasal applications are called for.

**Acetozone**, a new non-toxic germicide, is stated to be benzoyl-acetyl-peroxide, forming whitish crystals which melt at 98° F., and which undergo hydrolysis and slow decomposition in the presence of water. It is marketed in the form of a powder containing 50 per cent. of pure acetozone. It can be administered in capsules, diluted with sugar of milk, lycopodium, etc., or in solution by adding 25 grains to a quart of distilled water. This can be taken internally, or used as a spray or for irrigation, etc. As a dusting powder acetozone should be diluted with 100 to 500 parts of boric acid, talc, or other material; or 1 to 5 per cent. may be combined with petroleum ointment bases for use as an ointment. We have found it acceptable and well borne in gastro-enteritis. A pint of the solution drunk daily seemed to have excellent results as an internal antiseptic, but we cannot speak from personal experience of its use in typhoid fever, for which it is recommended administered in this manner.

**Terpin Hydrate and Heroin Co.** in chocolate-coated tablets is an agreeable form in which to prescribe this useful combination.

